# *Brachypodium* Genomics

**DOI:** 10.1155/2008/536104

**Published:** 2008-02-06

**Authors:** Bahar Sogutmaz Ozdemir, Pilar Hernandez, Ertugrul Filiz, Hikmet Budak

**Affiliations:** ^1^Biological Science and Bioengineering Program, Faculty of Engineering and Natural Sciences, Sabanci University Orhanli, 34956 Tuzla-Istanbul, Turkey; ^2^Institute for Sustainable Agriculture (IAS), Spanish National Research Council (CSIC), Alameda del Obispo s/n, Apartado 4084, 14080 Cordoba , Spain

## Abstract

*Brachypodium distachyon* (L.) Beauv. is a temperate wild grass species; its morphological and genomic characteristics make it a model system when compared to many other grass species. It has a small genome, short growth cycle, self-fertility, many diploid accessions, and simple growth requirements. In addition, it is phylogenetically close to economically important crops, like wheat and barley, and several potential biofuel grasses. It exhibits agricultural traits similar to those of these target crops. For cereal genomes, it is a better model than *Arabidopsis thaliana* and *Oryza sativa* (rice), the former used as a model for all flowering plants and the latter hitherto used as model for genomes of all temperate grass species including major cereals like barley and wheat. Increasing interest in this species has resulted in the development of a series of genomics resources, including nuclear sequences and BAC/EST libraries, together with the collection and characterization of other genetic resources. It is expected that the use of this model will allow rapid advances in generation of genomics information for the improvement of all temperate crops, particularly the cereals.

## 1. INTRODUCTION

*Brachypodium* P. Beauv (from the Greek *brachys* “short” and *podion* “a little foot,” referring to its subsessile spikelets, [1]) is a genus representing some temperate wild grass species. In particular, *Brachypodium distachyon* (L.) Beauv., also described as “purple false broom,” has recently emerged as a new model plant for the diverse and economically important group of temperate grasses and herbaceous energy crops [2]. Temperate crops such as wheat, barley, and forage grasses are the basis for the food and feed supply. However, the size and complexity of their genomes are major barriers to genomics research and molecular breeding. Similarly, although the herbaceous energy crops (especially grasses) are becoming novel target sources of renewable energy, very little is known about the biological basis underlying their bioenergy traits. Therefore, there is a growing need for a temperate grass model to address questions directly relevant both for improving grain crops and forage grasses that are indispensable to our food/feed production systems, and for developing grasses into superior energy crops. The present status of genomics research conducted in this model grass species is briefly summarized in this review.

## 2. *BRACHYPODIUM* GENOMICS AS A MODEL SYSTEM

### 2.1. Desirable attributes

**Figure 1:**
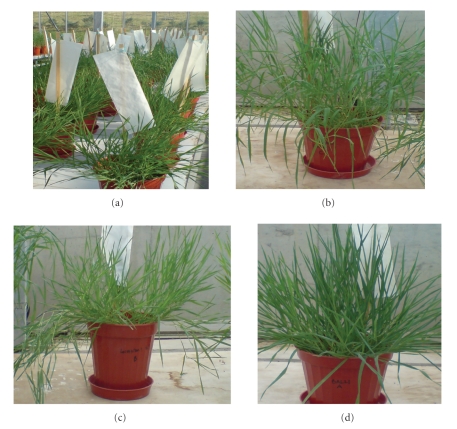
The *Brachypodium distachyon* lines grown under greenhouse conditions. (a) Seed heads are covered to prevent crosspollination in case it persists. (b), (c), and (d) seeds were collected from a diverse geographic region of Turkey.

*B. distachyon* has many attributes that make it a suitable model for conducting functional genomics research not only among cereal crops like wheat and barley, but also for biofuel crops like Switchgrass [2]. Due to its small haploid genome (~355 Mbp) and availability of a polyploid series with a basic chromosome number of *x* = 5, (2*n* = 2*x* = 10), the diploid race of *B. distachyon* can be used as a model for the much larger polyploid genomes of crops such as bread wheat (16979 Mbp, 2*n* = 6*x* = 42), durum wheat (12030 Mbp, 2*n* = 4*x* = 28), and barley (5439 Mbp, 2*n* = 2*x* = 14) (all C-values from [3]). Besides its small genome size, other desirable attributes include a small physical stature (approximately 20 cm), self-fertility, lack of seed-shattering, a short lifecycle that is normally completed within 11–18 weeks depending on the vernalization requirement [2] (might be as fast as 8 weeks under optimized conditions, [4]), and simple growth requirements with large planting density and easy genetic transformation [4, 5]. This combination of desirable attributes, together with the biological similarities with its target crops, is responsible for the recent research interest in this species. A few ecotypes of this taxon collected from diverse geographic regions of Turkey are shown in Figure 1, indicating a high level of variation among different accessions.

*Brachypodium* species range from annuals to strongly rhizomatous perennials that exhibit breeding systems ranging from strictly inbreeding to highly self-incompatible [6]. Some of the characteristic features of the genus *Brachypodium* include the following [17]: (i) hairy terminal ovary appendage, (ii) the single starch grains, (iii) the outermost thick layer of the nucellus, (iv) the long narrow caryopsis, (v) spicate or racemose inflorescences, and (vi) hairy nodules [7].

### 2.2. Brachypodium as a model system: a comparison with Arabidopsis and Oryza

The available genome sequences of the model plants *Arabidopsis* [9] and rice [10] are considered to be the major resources for plant genomics research. Nevertheless, these model species are not suitable for the functional genomics studies of temperate grasses. *Arabidopsis* has all the desirable attributes for a model plant: it is small in size, grows easily and quickly (reaching maturity in 6 weeks), has a small diploid genome, is self-compatible and easily transformable. Its utility as a model system has been proven by the wealth of genomic discoveries, useful for a broad range of crops (including cereals) it has generated. However, as a dicot species, it does not share with grass crops most of the biological features related to agricultural traits and in this sense, rice would provide a better alternative. The rice plant, however, does not fulfill the requirements of short size, rapid life cycle, inbreeding reproductive strategy, simple growth requirements, or easy transformation, thus imposing practical limitations. As a tropical species, it does not display all agronomic traits that are relevant to temperate grasses, especially to forage grasses; these agronomic traits include resistance to specific pathogens, freezing tolerance, vernalization, perenniality, injury tolerance, meristem dormancy mechanisms, mycorrhizae, sward ecology, or postharvest biochemistry of silage [2]. Moreover, rice is phylogenetically distant from the *Pooidae* subfamily that includes wheat, barley, and temperate grasses [11], whereas *Brachypodium* diverged from the ancestral *Pooidae* clade immediately prior to the radiation of the modern “core pooids” (*Triticeae*, *Bromeae*, and *Avenae*), which include majority of important temperate cereals and forage grasses [8]. Based upon cytological, anatomical, and physiological studies, *Brachypodium* is placed into its own tribe *Brachypodieae* of the Poaceae family [12]. In fact, the perennial outbreeding species, *B. sylvaticum* (2*n* = 18) was considered suitable for study of archetypal grass centromere sequences, which allowed detection of repetitive DNA sequences that are conserved among wheat, maize, rice, and *Brachypodium* [13]. Several species of this genus were studied using combined sequences of chloroplast *ndh*F gene and nuclear ITS to reconstruct phylogeny among these selected species within the genus [8]. Similarly, RFLPs and RAPDs were used for nuclear genome analysis to establish the evolutionary position of the genus. The genus *Brachypodium* constitutes morphologically more or less closely resembling species that are native to different ecological regions such that *B. distachyon* is the Mediterranean annual, nonrhizomatous *B. mexicanum* is from the New World, *B. pinnatum* and *B. sylvaticum* are Eurasian, and *B. rupestre* is a European taxon [7]. In view of the above, *B. distachyon* has been proposed as an alternative model for functional genomics of temperate grasses [2].

### 2.3. *Brachypodium* and the tribe Triticeae

The tribe *Triticeae* Dumort belongs to the grass family, Poaceae, and constitutes one of the economically most important plant groups. It includes three major cereal crops, wheat, barley, and rye (belonging to the genera *Triticum*, *Hordeum*, and *Secale*, resp.), which are traditionally cultivated in the temperate zone. The tribe has basic chromosome number of seven, and contains taxa ranging from diploids (2*n* = 2*x* = 14) to duodecaploids (2*n* = 12*x* = 84), including all intermediate ploidy levels [14]. Polyploidy (the most important cytogenetic process in higher plants, [15]) and more specifically, allopolyploidy, has played and plays a main role in the tribe’s evolution. With around 350 species, crossability barriers are poorly understood, and it is remarkable how its species, even species in different genera, can be made to hybridize even if they do not hybridize naturally. Therefore, the tribe also includes man-made crops such as × *Triticosecale* (triticale) and × *Tritordeum* (an amphiploid of *Triticum aestivum* × *Hordeum chilense* [16, 17] and *Triticum aestivum* × *Leymus arenarius* [18]). It has also been possible to apply interspecific and intergeneric hybridization to increase the genetic variability of crops belonging to this tribe (mainly wheat, [19, 20]).

As mentioned earlier, the chromosome numbers within *B. distachyon* accessions range from 10 to 30 [21], and the haploid genome size in diploid *Brachypodium* (2*n* = 2*x* = 10) varies from 172 Mbp to 355 Mbp [2, 22], although the former value may be an underestimate [22]; the genome size is thus assumed to be approximately 355 Mbp. Therefore, within Poaceae, *B. distachyon* carries one of the smallest genomes, which is intermediate between the genomes of *Arabidopsis thaliana* with 157 Mbp, and rice with 490 Mbp (All C-values from [3]). These data are consistent with previous reports that species of *Brachypodium* have the smallest 5S rDNA spacer among the grasses, and contain less than 15% highly repetitive DNA [7]. Additionally, GISH analysis of somatic chromosomes has shown preponderance of repetitive DNA in the pericentromeric regions, reflecting the compactness and economy of this genome [12]. This study also revealed structural uniformity of the diploid accessions ABR1 and ABR5, confirming their status as model genotypes, from which two BAC libraries have recently been prepared for functional genomics analysis [23]. Recent analysis of BAC end sequences (BESs) also corroborates this unusually compact genome [24, 25]. The other accessions having chromosome numbers in multiples of 10 suggested that this species has evolved as a polyploid series based upon 2*n* = 2*x* = 10, and that ecotypes that deviate from multiples of 10 evolved due to aneuploid or dysploid changes in chromosome number [21]. A cytotaxonomic analysis of the members of the polyploid series has revealed hybrid origin of several of the polyploid genotypes, suggesting a complex evolution of this species that is not entirely based on chromosome doubling [12]. For example, allotetraploid artificial hybrids between *B. distachyon* and *B. sylvaticum* exhibited irregular meiosis and infertility [6], although allotetraploids were fertile, one of them (ABR100) showed normal meiosis [12]. This indicates that either the constituent diploids of this allotetraploid are more compatible in hybrids, or the hybrids themselves have evolved pairing control mechanisms similar to those of wheat and other allopolyploids. It has also been shown using GISH that the genomes of the constituent diploids remain separated in the allotetraploid, and that there is no recombination between homoeologous chromosomes. These features make the natural polyploid hybrids within the genus *Brachypodium* a suitable material for the isolation and characterization of diplodizing genes.

For the reasons stated above, the whole tribe Triticeae is considered to be an enormous gene pool for crop improvement, deserving efforts not only for its morphological, physiological, genetic, and genomic characterization, but also for the establishment of phylogenetic relationships among different species of the tribe. The large and complex genomes of some members of the tribe are a main constraint for genomics research within this tribe, which would be greatly facilitated with the availability of a suitable model species like *B. distachyon*.

## 3. CURRENT STATUS OF *BRACHYPODIUM* GENOMICS

### 3.1. Development of inbred lines

Inbred lines make an important resource for genomics research. Keeping this in view, diploid inbred lines have been developed in *B. distachyon* by selfing [4] as well as through selection from segregating populations derived from crosses among diploid ecotypes [26].

### 3.2. Development of transformation and regeneration protocols

**Figure 2:**
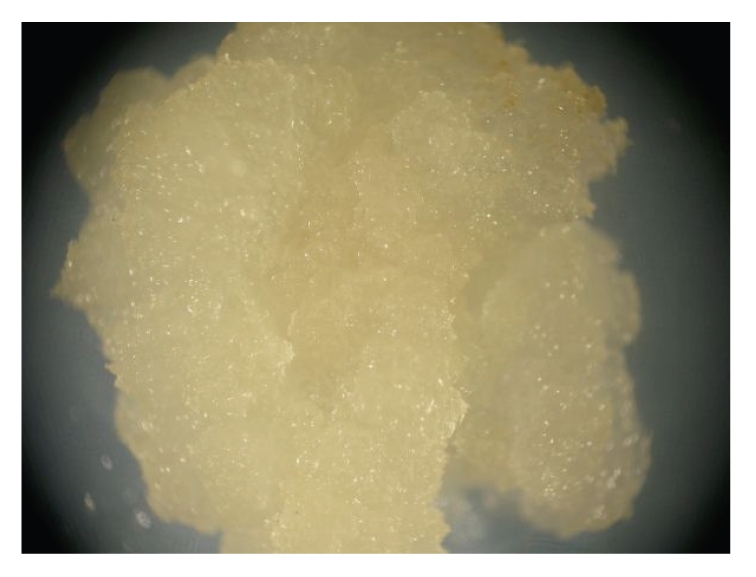
Callus induction of mature *Brachypodium distachyon* embryos supplemented with 5 mg/L 2,4-D (2,4-dichlorophenoxyacetic acid).

An efficient transformation procedure and an optimized plant regeneration protocol have been developed in *B. distachyon*. For instance, in a study reported in 1995, callus induction and plant regeneration from mature embryos, as well as *in vitro* clonal propagation of shoots were successfully achieved in *B. distachyon* [27]. In our own studies also, efficient callus formation from mature embryos of *B. distachyon* was successfully achieved using MS basal medium supplemented with sucrose and 2,4-dichlorophenoxyacetic (2,4-D) acid at a concentration ranging from 2.5 to 5 mg\L (see Figure 2). The results are suggesting that in *Brachypodium* species, higher rates of callus induction can be achieved through the use of (i) MS basal medium [43] rather than LS basal medium [44] (ii) sucrose rather than maltose, and (iii) higher concentrations of auxin as a plant growth regulator (unpublished data).

*Agrobacterium*-mediated transformation involving insertions of single genes has also been achieved in several genotypes of *B. distachyon* (including diploid and polyploid taxa), giving T_1_ transgenic plants [4]. These transformation studies also involved the diploid genotype Bd21, which has also been used for construction of BAC libraries [28] and for generating 20440 ESTs [29]. *Agrobacterium*-mediated transformation was successful in 10 out of the 19 lines, with efficiencies ranging from 0.4% to 15% [4]. Embryogenic calli derived from immature embryos were also transformed through biolistic transformation leading to transgene expression in T_1_ progeny [2, 5]. In later study, transformation with an average efficiency of 5.3% was achieved. In this study, testing of T_0_ as well as T_1_ generations and seed production in T_2_ was achieved within one year due to the short life cycle of *B. distachyon* [5], confirming its importance as a model plant species.

### 3.3. BAC libraries and expression sequence tags (ESTs)

BAC libraries of two diploid ecotypes of *B. distachyon*, ABR1 and ABR5, have also been constructed and have been used to determine synteny among rice, *Brachypodium*, and other species of Poaceae family. For this purpose, BACs were marker-selected (BAC landing) using primers designed according to previously mapped rice and Poaceae sequences. Most BACs hybridized as single loci in known *Brachypodium* chromosomes, whereas contiguous BACs colocalized on individual chromosomes, thus confirming conservation of genome synteny [23].

### 3.4. Mutagenesis

Mutagenesis with sodium azide was also successful in *B. distachyon* although response to this mutagen differed among different accessions [30]. The results obtained were comparable with those earlier obtained in barley and rice under higher concentrations of mutagens. Application of ethylmethane sulphonate (EMS) is currently on the way in diploid *Brachypodium* accessions.

## 4. *BRACHYPODIUM* GENOMES: ADVANCES ON THE WAY

### 4.1. BAC-based physical maps

A BAC-based physical map of *B. distachyon* is being developed at the John Innes Centre (Norwich, UK) as an aid to the international effort to make BAC-based physical maps of the genomes of Chinese Spring bread wheat [31]. Since establishing a physical map of the genome of bread wheat, one of the most important crops worldwide, is a major challenge due to the enormous size of the genome and its hexaploid constitution, it is expected that the availability of a *Brachypodium* physical map will greatly facilitate this task. The close phylogenetic relationship of *Brachypodium* to wheat leads to high similarity in gene sequences. Unambiguous hybridization signals are also generated, when *Brachypodium* probes are used on wheat BAC filters and southern blots. Preliminary experiments have also shown that it is feasible to anchor *Brachypodium* BACs to the rice genome by BES to create an outline physical map. An outline physical map of *B. distachyon* genotype, Bd3-1 using BES and fingerprinting, is being established and will be used to start assembling contigs in wheat chromosome groups [31].

Another *B. distachyon* physical map is being developed at the University of California and US Department of Agriculture (USDA) [25] by using two BAC libraries constructed from *B. distachyon* genotype, BD-21. These BACs are being fingerprinted using snapshot-based fingerprinting. This physical map of *B. distachyon* will also be integrated with BES, again providing genome-wide *Brachypodium* resources for sequence assembly, comparative genome analysis, gene isolation, and functional genomics analysis.

### 4.2. B. distachyon genome and retrotransposons

The genome of *B. distachyon* is also being examined for the presence, diversity, and distribution of the major classes of plant transposable elements, particularly the retrotransposons [32], since retrotransposons comprise most of the existing DNA between genes in the large cereal genomes. The compact genome of *B. distachyon* contains relatively few retrotransposons, which include copia, gypsy, TRIM, and LARD groups of elements. The availability of retrotransposon sequences will facilitate the development of retrotransposon-based molecular markers like IRAP, REMAP, SSAP, and RBIP markers, which have a variety of applications.

### 4.3. Genetic linkage maps

A genetic map of *B. distachyon* genotype, Bd21 is being developed by the International *Brachypodium* Initiative [33]. Genetic maps will provide anchor points linking the genome of *Brachypodium* with those of rice, wheat, and some biofuel crops, and will establish chromosome-scale physical maps of BACs for whole genome sequencing. In order to develop these genetic maps, mapping populations are being developed, which currently comprise several hundred F_2_ lines derived from the cross Bd21 × Bd3-1. These will be advanced to F6 to establish RILs that can serve as a common mapping resource for the community. Several approaches have been used to identify polymorphisms between parents of the mapping population. First, conserved orthologous sequence (COS) markers derived from wheat and millet were used to identify a set of 80 confirmed polymorphisms between these two parental lines (Bd21, Bd3-1). Another strategy was the use of ESTs derived from Bd21 in order to identify additional polymorphisms [29]. The most productive approach has been to predict introns in *Brachypodium* genes, based on a comparison of Bd21 ESTs with the annotated rice genome sequence; nearly all primers designed from predicted introns gave amplified products in PCR reactions. Most markers developed thus were polymorphic among the 5 diploid inbred lines used for testing, and thus proved to be useful markers for genetic mapping.

### 4.4. Whole genome sequencing

The *Brachypodium* nuclear genome is currently being sequenced within a project that was funded in early 2006 by the US Department of Energy (DOE). A draft genome sequence is expected to be completed by the end of 2007. This project is generating a whole-genome shotgun sequence of *B. distachyon* genotype, Bd21 genome, and is coupled with another project aimed at generating nearly 250.000 ESTs. Data from both projects will be made publicly available through an online database (BrachyBase at http://www.brachybase.org) and a community-dedicated portal (http://www.brachypodium.org). BrachyBase will enable efficient exploitation of genome and transcriptome sequences to identify genes underlying traits and will facilitate comparisons with other grass genomes [34].

Generation and analysis of over 60 000 BES from large-insert BAC clones has provided the first view of *Brachypodium* genome composition, structure, and organization [35]. In this study, ~10% of the BES show similarity to known repetitive DNA sequences in existing databases, whereas ~40% matched sequences in the EST database, which suggests that a considerable portion of the *Brachypodium* genome is transcribed. Gene-related BESs that were identified for the *Brachypodium* genome were also aligned *in silico* to the rice genome sequences. On the basis of gene colinearity between *Brachypodium* and rice, conserved and diverged regions were identified. BES with significant matches to wheat ESTs that have been mapped to individual chromosome and bin positions were also identified. These BACs represent regions that are colinear with mapped ESTs and will be useful in identifying additional markers for specific regions of wheat chromosomes.

A 371-kb region in *B. sylvaticum* has already been sequenced was also compared with orthologous regions from rice and wheat genomes [36]. In this region, *Brachypodium* and wheat showed perfect macrocolinearity, whereas rice contains an approximately 220-kb inversion. Using conserved genomic and EST sequences, divergence between *Brachypodium* and wheat was estimated to be 35–40 million years, which is significantly more recent than the divergence of rice and wheat, which is estimated to have occurred approximately 50 million years [37].

Chosen target loci from *Brachypodium* genome are also being sequenced and compared with genomic sequences from a variety of plant species including the following: (i) wheat species (*Triticum* and *Aegilops*) with different ploidy levels, (ii) rice, and (iii) *B. sylvaticum*, for which a BAC library is available [38, 39]. This comparison revealed that there is a better conservation of microcolinearity between wheat and *Brachypodium* orthologous regions than between wheat and rice, as was also shown in an earlier study [36]. For instance, sequence comparison at the grain hardness locus shows that genes responsible for grain hardness/softness, which is seed quality trait in wheat, are absent from the rice orthologous region, but present in the *B. sylvaticum* orthologous region. The gene density found in *B. sylvaticum* genome is comparable to that of rice (one gene per 8 kb). These results illustrate that *Brachypodium* species may represent an intermediate model for wheat genome analysis.

To test the potential of *Brachypodium* as a model for the functional analysis of ryegrass (*Lolium perenne*) flowering genes, expression of two *Terminal Flower* 1 orthologs, namely, *LpTFL* 1 (from *L. perenne*) and *TFL* 1 (from *Arabidopsis*), was examined in two different *B. distachyon* accessions [40]. Both these repressors significantly delayed heading date. The short life cycle of *Brachypodium* and the rapid transformation system allowed heading date scoring of T1s within the first year after transformation, thus demonstrating the potential of *Brachypodium* as a model for ryegrass (*L. perenne*) also.

*Brachypodium* is also being explored as a model for the genomics research involving study of cereals-pathogen interactions. For instance, varying degrees of susceptibility and resistance to *Magnaporthe grisea* (economically destructive pathogen and casual agent of Rice Blast disease that can also infect temperate cereals and forage grasses) have been found in several *Brachypodium* accessions. Aetiology of fungal development and disease progression in *Brachypodium* closely resembled those of rice infections; an overexpression of genes that were homologous with barley genomic probes was also observed [41]. Recent advances in *Brachypodium* genomics also involved use of metabolic profiling using Fourier-transform infrared spectroscopy (FT-IR) for high-throughput metabolic fingerprinting and electrospray ionization mass spectrometry (ESI-MS). These metabolomic approaches have shown considerable differential phospholipids processing of membrane lipids during *M. grisea-B. distachyon* accessions ABR1 (susceptible) and ABR5 (resistant) interactions [42]. *Brachypodium distachyon,* being a host for *M. grisea* and other disease-causing pathogens of Pooid cereals [42], is a suitable model for conducting functional genomics research involving study of *M. grisea* pathology and plant responses [41].

## 5. CONCLUSIONS

With the small genome size and simple growth requirements, *Brachypodium* provides us with a genome, which is a model for in-depth understanding of functional genomics of temperate grass genome. As a model, it overcomes some of the drawbacks that are inherent in the genomes of *Arabidopsis* and rice that have already been sequenced and have been hitherto considered models for the improvement of crop species like wheat and barley. Therefore, elucidation and an improved understanding of *Brachypodium* genomics has enormous potential to benefit all phases of society. It provides improved, efficient, and effective genetics and genomics program. The knowledge on *Brachypodium* genome is also useful for an in-depth understanding of evolutionary relationships among different plant genomes. This will play a pivotal role in comparative studies in diverse fields such as ecology, molecular evolution, and comparative genetics.
